# Microbial-derived products as potential new antimicrobials

**DOI:** 10.1186/s13567-018-0563-5

**Published:** 2018-07-31

**Authors:** Bruce S. Seal, Djamel Drider, Brian B. Oakley, Harald Brüssow, David Bikard, Joseph O. Rich, Stefan Miller, Estelle Devillard, Jason Kwan, Gérard Bertin, Stuart Reeves, Steven M. Swift, Margot Raicek, Cyril G. Gay

**Affiliations:** 1 Biology Program, Oregon State University Cascades, 1500 SW Chandler Avenue, Bend, OR 97702 USA; 2 Institut Charles Viollette, Université Lille 1, 59000 Lille, France; 3 College of Veterinary Medicine, Western University of Health Sciences, 309 E Second St, Pomona, CA 91766-1854 USA; 4 Nestlé Research Centre, Nestec Ltd, Vers-chez-les-Blanc, 1000 Lausanne 26, Switzerland; 5 Synthetic Biology Group, Microbiology Department, Institut Pasteur, 75015 Paris, France; 6 Renewable Product Technology Research Unit, National Center for Agricultural Utilization Research, Agricultural Research Service, U.S. Department of Agriculture, 1815 North University Street, Peoria, IL 61604 USA; 7 Lisando GmbH, Josef-Engert-Straße 13, 93053 Regensburg, Germany; 8 Nutrition Research Team, Adisseo France S.A.S.-CERN, 6 Route Noire, 03600 Commentry, France; 9 School of Pharmacy, University of Wisconsin, 777 Highland Ave., Madison, WI 53705-2222 USA; 10 European Probiotic Association & Erawan Consulting SARL, Asnières Affaires, 25 rue des Bas, 92600 Asnières-sur-Seine, France; 11 Embria Health Sciences, 2105 SE Creekview Dr., Ankeny, IA 50021 USA; 12 Animal Biosciences and Biotechnology Laboratory, BARC, Agricultural Research Service, USDA, 10300 Baltimore Ave, Beltsville, MD 20705-2350 USA; 13 Intern, World Organisation for Animal Health (OIE), 12 rue de Prony, 75017 Paris, France; 14 National Program Staff-Animal Health, Agricultural Research Service, US Department of Agriculture, Beltsville, MD 20705 USA

## Abstract

Due to the continuing global concerns involving antibiotic resistance, there is a need for scientific forums to assess advancements in the development of antimicrobials and their alternatives that might reduce development and spread of antibiotic resistance among bacterial pathogens. The objectives of the 2^nd^ International Symposium on Alternatives to Antibiotics were to highlight promising research results and novel technologies that can provide alternatives to antibiotics for use in animal health and production, assess challenges associated with their authorization and commercialization for use, and provide actionable strategies to support their development. The session on microbial-derived products was directed at presenting novel technologies that included exploiting CRISPR-Cas nucleases to produce sequence-specific antimicrobials, probiotics development via fecal microbiome transplants among monogastric production animals such as chickens and mining microbial sources such as bacteria or yeast to identify new antimicrobial compounds. Other research has included continuing development of antimicrobial peptides such as newly discovered bacteriocins as alternatives to antibiotics, use of bacteriophages accompanied by development of unique lytic proteins with specific cell-wall binding domains and novel approaches such as microbial-ecology guided discovery of anti-biofilm compounds discovered in marine environments. The symposium was held at the Headquarters of the World Organisation for Animal Health (OIE) in Paris, France during 12–15 December 2016.

## Introduction

Due to global concerns of increasing antimicrobial resistances, at the request of the National Institute of Allergy and Infectious Diseases of the US National Institutes of Health, the National Research Council (NRC) of the US National Academy of Sciences organized two workshops during 2006 to coordinate discussions on approaches for developing new antimicrobial therapeutics and for development of immunomodulatory methods for treatment of infectious diseases [1]. The NRC committee understood that most antibiotics are natural products produced by microorganisms themselves as secondary bioactive metabolites that render susceptible neighbors inactive, but also recognized that those competing microorganisms develop many resistance strategies [2]. Consequently, the NRC committee recommended improving diagnostics for continued surveillance of antibiotic resistance microorganisms, accompanied by development of strategic antimicrobials that selectively target specific pathogens to avoid dysbiosis caused by broad-spectrum antibiotics. Furthermore, recommendations also included a further need to determine the composition of normal resident microbiota and understand the relationship between those resident microbes relative to host health.

Antibiotics have been vital in combating disease-causing bacteria for more than 80 years since the discovery and large-scale production of penicillin [3]. During that time, many different antimicrobials have been developed, however despite the evolution of antimicrobial resistances there has been little recent commercial marketing of new antimicrobials [4]. Consequently, a variety of approaches have been recommended to investigate new alternatives that could potentially accomplish some functions of traditionally utilized antibiotics. The principle NRC recommendations as stated are a need to characterize the normal resident microbes in the host, determine bacterial mechanisms that can be used in pre/pro-biotic therapies, identify effective delivery mechanisms and develop strategies that will selectively target pathogenic organisms [1]. Finding alternatives would be valuable to food-animal production, where the use of antimicrobial growth promoters (AGPs) in animal feeds has been implicated in development of resistance mechanisms in bacteria [5]. Concerns about overuse and misuse of antibiotics in animal production led to a ban on antibiotics for use as growth promoters that began 1 January 2006 in the European Union (EU) [6]. At the international level, organizations such as the World Health Organization, the Food and Agricultural Organization of the United Nations, and the World Organisation for the Health of Animals (OIE) endorsed the Global Action Plan on Antimicrobial Resistance during 2015, which among other objectives aims to reduce antibiotic consumption in humans and animals [7].

The need to address the issues of antibiotic resistance among bacterial pathogens that pose a threat to both human and animal health and concerns over the misuse of antibiotics has garnered global interest in limiting antibiotic use among different biomedical and agricultural sectors. These concerns and alternatives were presented during the first “Alternatives to Antibiotics” (ATA) Symposium held during September 2012 at the World Organisation for Animal Health (OIE) in Paris. The symposium highlighted promising research results and novel technologies that could potentially lead to alternatives to conventional antibiotics, assess challenges associated with their commercialization, and provide actionable strategies to support development of new antimicrobials [8]. Subsequently, a second Alternatives to Antibiotics Symposium was held during December 2016 focusing on the latest scientific breakthroughs or technologies that could provide options and alternative strategies for preventing and treating diseases of animals [9] that could support a One Health approach [10]. This review summarizes presentations given during the Microbial-derived Products session wherein several topics were discussed including novel use of the CRISPR/cas system to act as sequence-specific antimicrobials, probiotics development via fecal transplantation, microbial-ecology driven discovery of antibiofilm compounds and other traditional sources of discovering novel antimicrobial compounds from microbial sources.

## Use of the CRISPR/cas system to develop novel antimicrobials

**Figure 1:**
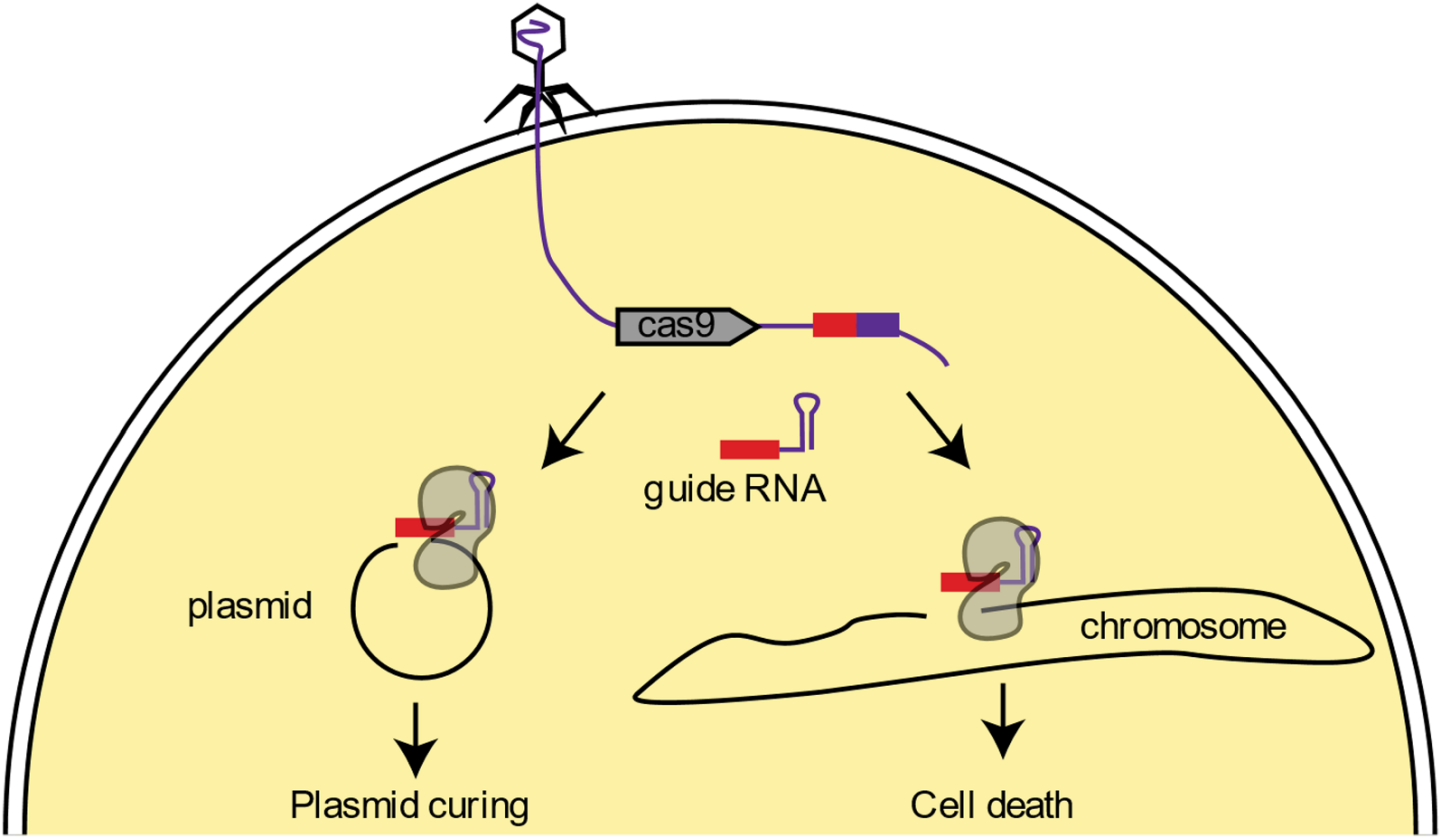
**The CRISPR system as an antimicrobial.** A phage vector is used to inject a CRISPR system in a population of target bacteria. The Cas9 RNA-guided nuclease is expressed together with a guide RNA that will direct it to cut a target sequence. When the target is carried on a plasmid, the plasmid is cured, possibly leading to re-sensitization to an antibiotics. When the target is carried on the chromosome, cells die as a result of the chromosome degradation.

Bacteria have genomic DNA designated CRISPR or clustered regularly interspaced short palindromic repeats, that are an array of short repeated sequences separated by spacers with unique sequences often derived from bacteriophages [11, 12]. CRISPR loci encode an adaptive immune system that is capable of capturing pieces of DNA from phages and integrate them as new spacers in the CRISPR array (Figure 1). The CRISPR array can then be transcribed and processed into short CRISPR RNAs (crRNAs) that guide CRISPR-associated (Cas) nucleases to destroy target nucleic acids. Discovery of these RNA-guided nucleases in the CRISPR system has led to many biotechnological applications and these systems are now widely used for genome editing applications. Recently two studies also demonstrated how the Cas9 protein can be directed to make “cuts” in the chromosome of bacteria and kill them in a sequence-specific manner [13, 14]. Because antibiotics act in a relatively indiscriminate manner impacting both pathogenic and commensal bacteria, there is a need for novel antimicrobials that selectively target specific pathogens to avoid reducing potentially beneficial microbes [2]. Consequently, the concept of CRISPR-Cas systems as programmable antimicrobials could be utilized in both heterologous and endogenous systems to selectively kill specific pathogenic bacterial species and strains [15]. However, delivery systems are required for this system to be employed as an antimicrobial, which has recently been reported by two research groups utilizing bacteriophage packaging systems as vectors to deliver CRISPR/cas antimicrobials [16–18].

The CRISPR/Cas system has been exploited to produce sequence-specific antimicrobials utilizing delivery by phage capsids as phagemids [16]. Bikard et al. [16] demonstrated that Cas9 can be re-programmed to target virulence genes to specifically kill virulent, but not avirulent, *Staphylococcus aureus* when the target gene is present in the chromosome. This is accomplished by re-sensitizing the population of bacteria to an antibiotic. The approach is particularly important because methicillin- and vancomycin-resistant *S. aureus* (MRSA and VRSA, respectively) are extremely difficult to treat, resulting in life-threatening infections that without effective antimicrobial therapy may require surgery to remove infected tissue [19].

The basic approach was to insert the *Streptococcus pyogenes* cas9, tracrRNA (trans-activating crRNA) and a minimal CRISPR array optimized for one-step cloning of crRNA sequences, into the staphylococcal vector pC194 generating a unique plasmid targeting the kanamycin resistance gene *aph*-*3*. Although this construct can be transformed into *S. aureus*, a phagemid capable of being replicated like a plasmid as a single-stranded DNA in viral particles, was developed by the investigators that also contained packaging genes and the packaging site for the staphylococcal ΦNM1 phage for phagemid transduction of the bacterium. It was determined that sequence-specific treatment was better than a non-specific, traditional antibiotic in limiting the incidence of antibiotic resistant bacteria. Consequently, a phagemid was constructed that targeted the *mecA* methicillin resistance gene to reduce MRSA strains from a mixed population of bacteria and then was successfully utilized to reduce a clinical MRSA isolate of *S. aureus.* The ultimate test was in a mouse skin MRSA colonization model wherein Bikard et al. [16] demonstrated a decrease in the proportion of MRSA cells from 50 to 11.2% that was significantly different from all the other treatment conditions including treatment with streptomycin that decolonized the mice of all staphylococci. Although there are specific issues to address, such as supplying sufficient numbers of phagemids during an active infection to control a bacterial pathogen, the system does provide for selective killing of a pathogen. This is accompanied by the multiplex nature of CRISPR-Cas systems that could be exploited to target several different species at the same time and/or several sequences of the same bacterium to prevent the rise of resistant mutants.

Agricultural or environmental applications of the CRISPR/Cas system certainly will play major roles, hopefully by improving technologies in these fields. Targeted genome editing may potentially play roles in improving animal production by subjecting fibroblasts to nuclear genome editing, followed by somatic cell nuclear transfer, resulting in live-born, food-producing animals carrying single-gene directed mutations [20]. Currently however, most research has been devoted to genome engineering of food crops to potentially improve their resistances to pests or to enhance nutritional value and increase their ability to grow on marginal lands [21]. There will certainly be ethical considerations for the use of CRISPR/Cas gene editing during agricultural production. Recently, the US Food and Drug Administration (FDA) has proposed that genome edited animals be treated as transgenics during the approval process, despite having approved the use of recombinant human antithrombin, the first ever therapeutic protein from genetically altered goats [22], suggesting that this may lead to future use of other gene editing systems for practical applications [23].

## Bacteriophages and lysins

Bacteriophages, viruses that infect bacteria, have been utilized as treatments to control bacterial infections as early as the 1920–1930’s and continues today in Eastern Europe. Additionally, bacteriophages have played major roles during seminal investigations of molecular biology and improvements for biotechnology applications [24]. Approaches to address antimicrobial resistance among animals and humans have included passive immunization with bovine milk antibodies, use of probiotics, prebiotics and bacteriophage therapy. Antibodies isolated from colostrum milk of dairy cows hyperimmunized with diarrhea-associated pathogens showed oral treatment efficacy in children hospitalized with rotavirus [25], but not with *E. coli* diarrhea. Conversely, in children a *Lactobacillus paracasei* ST11 probiotic showed treatment efficacy against bacterial, but not viral diarrhea [26]. Bovine milk oligosaccharides used as a prebiotic food supplement in bottle-fed infants induced a stool microbiota shift to a *Bifidobacterium*-dominated microbiota typically found in breastfed infants, raising the prospect of microbiota modulation with nutritional interventions [27]. Finally, a T4-like coliphage cocktail was developed at the Nestlé Research Center (NRC) in Lausanne and subsequently tested for safety in healthy children by the International Center of Diarrhoeal Diseases Research, Bangladesh [28]. This T4-like phage cocktail was assayed in parallel to a commercial Russian phage cocktail [29] in children hospitalized with acute *E. coli* diarrhea. No treatment effect over standard care (oral rehydration solution supplemented with zinc) was observed during the trial [30]. The treatment failure was tentatively explained by the fact that only 70 per cent of the children showed a microbiologically confirmed *E. coli* diarrhea, that pathogenic *E. coli* titers remained below the in vivo phage replication threshold and that only half of the children harbored *E. coli* susceptible to the phage cocktail [30]. In addition, the etiological role of major *E. coli* pathotypes in childhood diarrhea from developing countries was increasingly questioned in the scientific literature. When comparing a successful *Pseudomonas*-associated otitis externa infection with the unsuccessful *E. coli*-associated acute diarrhea phage therapy trial, several differences emerged leading to recommendations for future clinical trials. Based on this comparison, criteria of an infectious disease whereby phage therapy is more likely to be effective include (1) one should target an infection where a single pathogen with a high pathogenicity index causes a clinically easily diagnosed infectious disease; (2) the pathogen should be present at high titer and be physically accessible to the applied phage; and (3) chronic infections are better targets for phage therapy than acute infections. Practically, patients should be pre-screened for in vitro susceptibility to the phage. Also, prevention is easier than treatment with phages, while chronic infections are better targets than acute infections [31].

**Figure 2:**
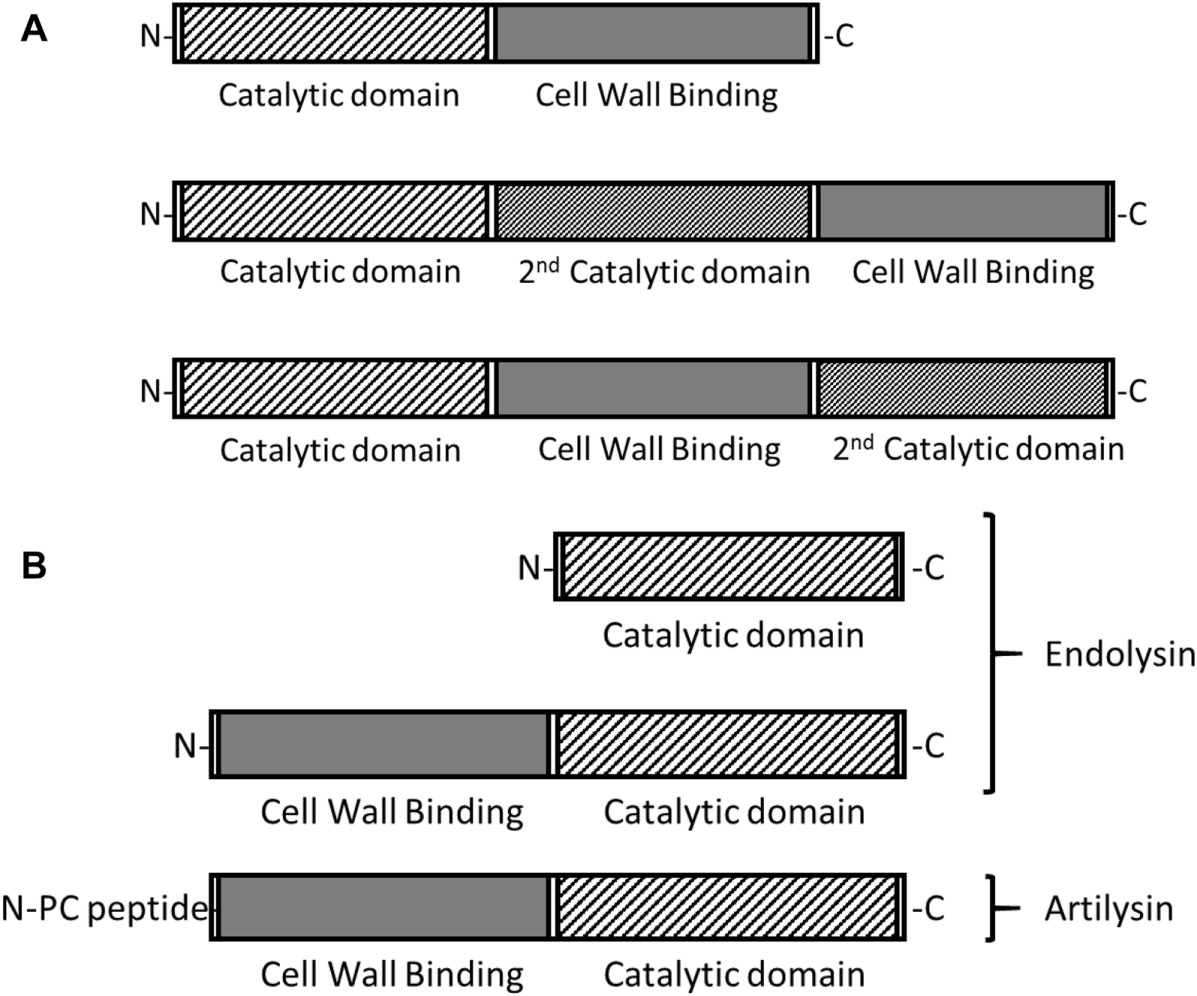
**Bacteriophage lysins as antimicrobials. A** Modular structure of Gram-positive bacteriophage endolysins. The typical endolysin against Gram-positive bacteria has a two-domain structure, an N-terminal catalytic domain and a C-terminal cell wall binding domain. Some endolysins incorporate a 2^nd^ catalytic domain with a catalytic mechanism different from the first catalytic domain. Targeting different parts of the peptidoglycan, catalytic domains can include l-alanine amidase, endopeptidase, muramidase, glucosaminidase, or lytic transglycosylase activities. Cell wall binding domains may contain single or multiple binding motifs. **B** Modular structure of Gram-negative bacteriophage endolysins, and of engineered Artilysins. The typical endolysin against Gram-negative bacteria has only a catalytic domain. Those Gram-negative endolysins with a cell wall binding domain have it located at the N-terminus of the protein. Gram-negative endolysins do not function when applied externally due to the presence of the outer membrane of Gram-negative bacteria. Engineered endolysins, called Artilysins, penetrate the outer membrane of Gram-negative bacteria through the addition of polycationic (PC) or hydrophobic/amphipathic peptide sequences to the N-terminus or C-terminus of the endolysin. Artilysins are effective when applied externally to Gram-negative bacteria.

Endolysins are bacteriophage-encoded muralytic enzymes that digest bacterial peptidoglycan exogenously (Figure 2). These recombinant proteins have been constructed as fusions of selected endolysins to specific outer membrane permeabilizing peptides, which promote transfer of the fusion protein across the outer membrane structure of Gram-negative bacteria [32]. Bacterial “persister” cells that are in a dormant metabolic state and do not replicate in the presence of antibiotics is a major component of the evolutionary response to antibiotics [33]. Therefore, making them resistant to most antibiotics that are active against replicating bacteria and making them capable of causing future clinical infection in a host [34]. Data has been published clearly demonstrating the ability of newly developed Artilysins to digest antibiotic resistant and non-replicating, persistent bacteria such as *Acinetobacter baumannii* [35] and *Pseudomonas aeruginosa* [36]. Currently, veterinary applications of these antimicrobials are reportedly being assayed for effective treatment of otitis and dermatitis bacterial infections [Miller S, unpublished].

Other bacteriophage and lysin technologies have been previously reviewed by others [37]. Recent attempts by investigators to induce prophage from *Staphylococcus aureus* bovine mastitis isolates as a potential to screen, identify and characterize bacteriophages, then potentially express their endolysins for use in non-antibiotic therapies of mastitis (Cullor J, U California, Davis, USA). Combinations of naturally occurring bacteriophages could potentially be administered early during the broiler chicken rearing period to control dysbacteriosis (Smyth VJ, Agri-Food and Biosciences Institute, Stormont Laboratories). Investigators at Synthetic Genomics in La Jolla, CA, USA have developed a cell-free phage engineering method that allows rapid and iterative editing of viral genomic DNA. They have engineered wide host-range phages to express biofilm degrading enzymes and antimicrobial moieties against *Pseudomonas aeruginosa* [38]. Bioinformatics was also utilized to analyze genomes of 43 unique *Clostridium perfringens* isolates from chickens and their genomes were searched for putative peptidoglycan hydrolase enzymes by homology to known lysins. There were several hundred putative peptidoglycan hydrolases identified and four proteins with homology to bacteriophage lysins were subsequently expressed and shown to have high lytic activity against all the *C. perfringens* chicken isolates as well as nine porcine isolates. Activity to lyse *C. perfringens* was demonstrated by plate lysis, zymograms and turbidity reduction assays [Donovan DM, ARS-USDA].

## Antimicrobial compounds from microbial sources

A variety of approaches can be utilized to discover bioactive compounds, specifically bioactive compounds to improve health and antimicrobials from a variety of microbial sources [39, 40]. There are a variety of novel antimicrobial compounds (Table 1) such as liamocin oil from the fungus *Aureobasidium pullulans* that has antibacterial activity with specificity for species of *Streptococcus* [41] and laparaxin, an antibacterial polypeptide secreted by *Lactobacillus paracasei* NRRL B-50314 that has antibacterial activity against a wide variety of Gram-positive bacteria [42]. Another focus has been on bacterial contaminants encountered during biofuel fermentations that inhibit ethanol production and how antibiotics are added to fuel ethanol fermentations [43]. Ethanol inhibition is most likely related to acetic acid production by contaminants, particularly by obligately heterofermentative species such as *Lactobacillus fermentum* and *L. mucosae*, requiring antibiotics such as virginiamycin to be utilized during the ethanol production process [44]. Consequently, in addition to liamocin and laparaxin, other non-antibiotic interventions such as bacteriophage lysins are being examined to resolve lactic acid bacterial contamination during ethanol-fuel fermentations with promising results [45].

**Table 1:** **Examples of alternative antimicrobials proposed to be utilized during ethanol fermentations as examples of novel approaches to replace traditional antibiotics**

Antimicrobial compound	Source	Target	Specificity	Agricultural/industrial production problem
Endolysins	Various bacteriophage	Gram positive bacteria	Narrow	Infections of industrial fermentations
Liamocin	*Aureobasidium pullulans*	*Streptococcus* species	Narrow	Mastitis, septicemia, neonatal mortality
Laparaxin	*Lactobacillus paracasei*	Gram positive bacteria	Broad	Food borne pathogens, drug resistant pathogens
Unknown	*Bacillus* sp.	*Lactobacillus* species	Narrow	Infections of industrial fermentations

Bacteriocins are ribosomally synthesized antimicrobial peptides produced by Gram-negative and Gram-positive bacteria. Many bacteriocins produced by food grade lactic acid bacteria (LAB) are safe and have activity against a narrow or a broad-range of bacteria as well as exert sporostatic/sporicidal activity against bacterial spores [46]. Meconium, the earliest stool of a mammalian newborn, contains different bacterial species such as *Enterococci*, *Bifidobacteria*, and *Lactobacilli* that protect mucus of infants from pathogenic species through production of antimicrobial substances [47]. Specifically, enterocins DD28 and DD93 produced by *E. faecalis* from meconium have methicillin-resistant *Staphylococcus aureus* (MRSA) bactericidal activity that have a synergistic effect in combination with erythromycin or kanamycin against a clinical MRSA-1 strain and inhibit biofilm formation by this bacterium [48]. Furthermore, a combination of leaderless enterocin DD14, colistin and nisin eradicated planktonic and biofilm cultures of *E. coli* CIP54127 and *E. coli* from swine with colistin-resistance phenotypes [49]. Consequently, bacteriocins can be utilized individually during food storage and bacteriocins from LAB could also be used synergistically as agents to augment antibiotic treatments. Microcin is a 21-amino acid polypeptide produced by *E. coli*, which has a unique lasso topology that confers stability [50]. At the ATA Conference it was reported that this peptide has bacteriostatic activity against *Salmonella* Newport ATCC 6962 and hence has promise as an antibacterial for members of the Enterobacteriaceae [51].

The lantibiotic nisin, a polycyclic antibacterial peptide, is produced by the bacterium *Lactococcus lactis* and a variant of *Streptococcus uberis.* It has broad-spectrum antibacterial activity and is used as an antimicrobial against many bacteria that are food-spoilage pathogens. Nisin was the first antimicrobial peptide approved for use as a food preservative; nisin European E number 234 [46]. Also reported was that nisin reduces *Mycobacterium avium* ssp*. paratuberculosis*, the associated Johne’s disease agent (paratuberculosis), in milk while the class IIa bacteriocin pediocin had no effect on this agent [Talaat AM, unpublished]. Nisin is also an effective inhibitor of the clostridia and can potentially be utilized to control *C. difficile* infections among humans since it is an effective inhibitor of both vegetative cells and spore germination [52]. *Lactococcus lactis* UL719 is a nisin Z producer that can inhibit *C. difficile* in a model of human colon [53]. Following nisin Z treatment, with a concomitant reduction of *C. difficile*, there was a decrease in short chain fatty acids with an alteration in the microbiota that returned to pre-treatment conditions after 24 h in the human colon model [52].

The possibilities of using natural products based on *Saccharomyces cerevisiae* fermentation with an in vitro mixed anaerobic culture system containing cecal microbiota has been used to aid in replacing antibiotics as growth promoters and improve gastrointestinal health among poultry by increasing short-chain fatty acid concentrations in the gut and decreasing preharvest levels of *Salmonella* Typhimurium [54]. Furthermore, addition of yeast fermentation products increased the microbial diversity of the chicken gastrointestinal tract significantly as the birds became older with *Ruminococcus*, *Faecalibacterium*, *Lachnospiraceae*, Clostridiales, and *Oscillospira* becoming the dominant bacterial groups [55]. Certain pathogens such as *Salmonella* released into the environment appear to be less virulent as judged by a human cell culture invasion assay and suppressed *hilA* expression [56].

## Fecal microbiome transplants and probiotics

Live microbial cultures or probiotics have been utilized for improving health of monogastric animals, however, new approaches should be taken to develop the “next generation” of novel therapeutic microbials for treatment of disease and improving animal health [57]. Fecal microbiota transplant (FMT) or fecal transplantation is a procedure in which fecal matter, or stool, is collected from a healthy donor, mixed with a physiological saline or similar solution, strained, and placed in a patient, by colonoscopy, endoscopy, sigmoidoscopy, or enema to restore a healthy microbiome to treat gastrointestinal disorders when practiced in humans [58]. The technique of rumen transfaunation, using the cud from a healthy donor animal to treat a sick recipient animal, was apparently applied long before any understanding of rumen microorganisms to treat indigestion of ruminants [59]. Although the practice is currently utilized for human patients, other than the report of rumen microbiota transplantation, this approach has not been utilized or further evaluated to discover new probiotics or to treat food-animals for gastrointestinal disorders and improve their microbiomes.

An innovative approach has been developed for probiotic development with fecal microbiome transplants as alternatives to antibiotics in broiler chickens (Oakley B, Western University). The approach entailed characterizing the gastrointestinal tract (GIT) microbiome of chicks from low- and high-efficiency genetic lines using high-throughput DNA sequencing. Secondly, groups of chicks from each genetic line received microbiome transplants from their own and the contrasting donor line to compare growth, feed efficiency, and effects on their microbiomes. Other experimentation included serially passaging GI tract contents that were transplanted to chicks and assaying for efficacy in resisting GI colonization of *Salmonella* spp. and *Campylobacter* spp. Results were promising, indicating that there were significant differences between high-efficiency inoculated versus uninoculated chicks as measured by body weight gain and feed efficiency that appeared to be mediated by the microbiota. Moreover, microbiome transplants also significantly improved pathogen resistance. Optimizing the microbiota of commercial poultry has potential to provide value to the industry by reducing feed costs, improving food safety, reducing the carbon footprint of the industry and potentially limiting regulatory burdens. This also provides an approach to identify new probiotic products as alternatives to antibiotics via comparative microbiome analyses with targeted cultivation.

The joint Food and Agriculture Organization (FAO) of the United Nations and World Health Organization (WHO) define probiotics as “live microorganisms which when administered in adequate amounts confer a health benefit to the host” and this is a widely-accepted definition adopted by the International Scientific Association for Probiotics and Prebiotics [60]. Probiotics, also known as direct fed microbials (DFMs), are widely utilized during food-animal production with the goal of promoting immune response and overall health or performance of livestock. The use of DFMs during food-animal production has increased over the past 25+ years and the official publication for the American Association of Feed Control Officials (AAFCO) lists 42 of these reagents acceptable as “food” products. The list of acceptable microbes includes *Lactobacillus* spp., *Bifidobacterium* spp., *Propionibacterium* spp., *Enterococcus* spp., *Pediococcus* spp., *Bacillus* spp. *Bacteroides* spp., along with the yeast *Saccharomyces cerevisiae* and two molds of the *Aspergillus* spp. [61]. Although these are the principle DFMs, probiotics may also include other microorganisms such as *Prevotella bryantii*, *Streptococcus* spp., *C. butyricum*, *E. coli*, *Lactococcus lactis*, *Megasphaera elsdenii* and *Candida pintolopseii* [60]. Interestingly, most commercial probiotic products assayed by high-throughput sequencing (HTS) most likely contain primarily *Lactobacillus acidophilus* and *Bifidobacterium animalis* subsp. *lactis* and this emphasizes the necessity for reliable methods to determine the taxonomy and quantify relative amounts of mixed microbial populations in commercial probiotic products [62].

Results have been reported using *Bacillus*-based probiotics [63], including investigating the immunomodulating properties of new *Bacillus subtilis* probiotic strains. Caco-2 cells in stimulated and non-stimulated conditions have been utilized to determine trans-epithelial resistance (TER) and IL-8 production as indicators of intestinal permeability and inflammation, respectively to select strains to improve poultry health [64]. They demonstrated that different *B. subtilis* strains can have different levels of efficacy in modulation of inflammatory response and intestinal permeability. Also, newly identified strains have potential to reduce intestinal inflammatory status and to enhance intestinal barrier qualities for improving food-animal performance in the absence of AGPs. *Bacillus*-based DFMs can have similar efficacy as bacitracin methylene disalicylate treatment to enhance broiler chicken performance during production [65]. *B. subtilis* addition improved gut microflora balance by leading to a significant increase in *Lactobacillus* and *Bifidobacterium* while significantly decreasing *Clostridium* spp. and coliforms (Kirwan S, Kemin). Probiotic inhibition of avian pathogenic *Escherichia coli* (APEC) by *Bacillus* strains administered during commercial broiler chicken production was reported and DFM *Bacillus* strains can inhibit bacterial pathogen adhesion and colonization of the mucosal surfaces in monogastric animals [64]. Utilizing an in vitro porcine cell-model it was reported that *Bacillus* spp. probiotics can aid in prevention of enterotoxigenic *E. coli* (ETEC) infection among swine (Cernat RC, Chr. Hansen).

*Lactobacillus* spp. are also commonly utilized probiotics [60, 61], and *L. casei* can reduce *Mycobacterium avium* ssp. *paratuberculosis* relative to the use of nisin as reported in the bacteriocin section (Talaat A, University of Wisconsin). It was reported that chitosan-based coating or use of probiotic *Lactobacillus* spp. reduces the presence of *Campylobacter* spp. on raw poultry products (Donoghue A, ARS-USDA) and *Lactococcus lactis* can be used as an intramammary infusion into the mammary glands of healthy mice to evaluate the potential use of this treatment for mastitis therapy [66].

**Figure 3:**
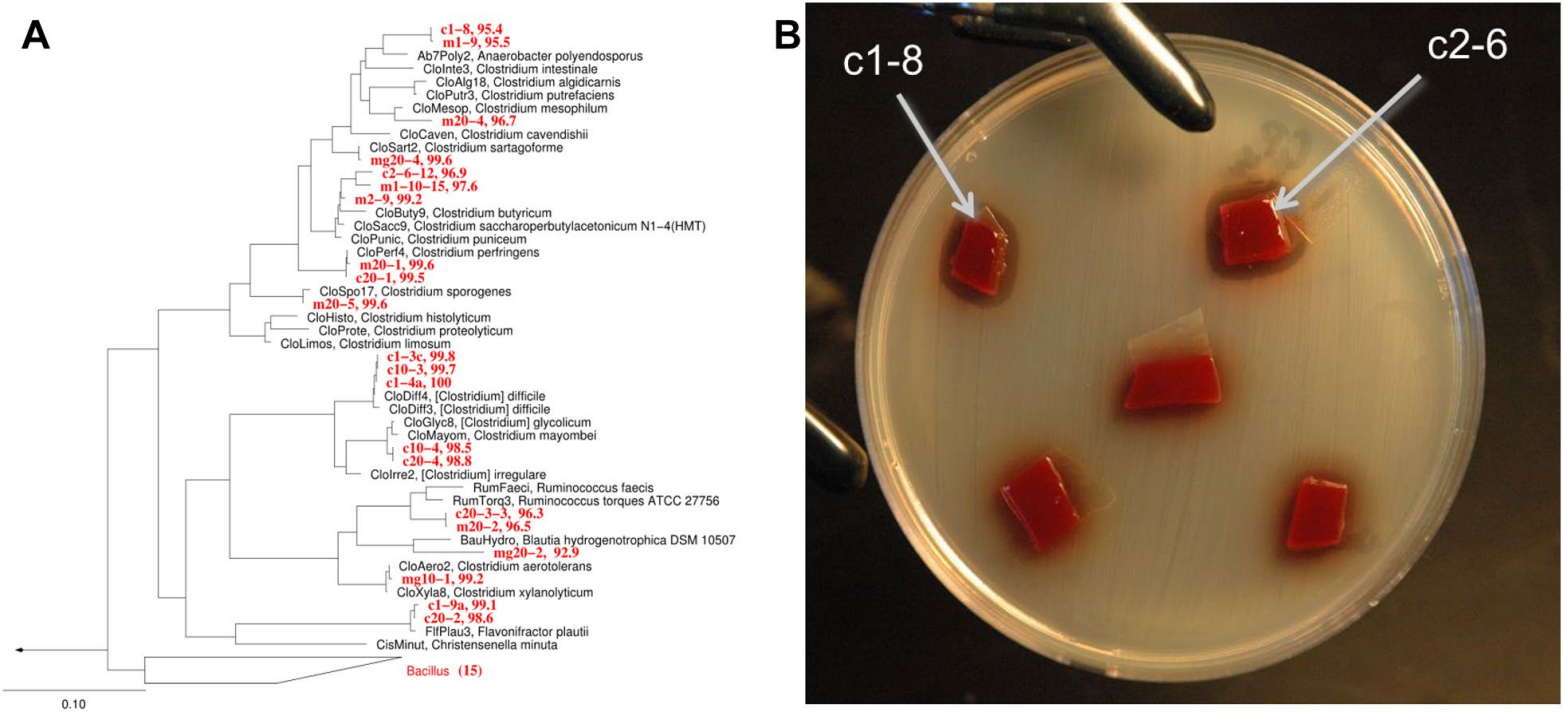
**Phylogenetics of newly identified potential clostridial probiotics and growth inhibition of** ***Clostridium perfringens*** **by these probiotic bacteria. A** 16S rRNA-based phylogeny of newly-isolated clostridia based on maximum-likelihood phylogenetic reconstruction of full-length (>1400 bp) 16S rRNA gene sequences. Taxa shown in red represent axenic cultures with representative nearest cultured isolates from v115 of the Silva database shown in black. Numbers after taxa represent % identity to closest cultured representative as determined by global usearch against a comprehensive reference database. Approximately 15 strains most closely related to *Bacillus* spp. were also isolated as indicated by the collapsed clade at the bottom of the figure. **B** Growth inhibition of *C. perfringens* by strains c2-6 and c1-8 (shown with arrows) demonstrated anti-microbial properties when placed on a lawn of *C. perfringens*. The mechanisms causing the underlying this phenomenon are still unknown.

Utilizing naturally occurring bacteria as probiotics has several advantages including the potential to compete against ecologically similar taxa and are potentially more likely to be approved by regulatory agencies for use during food-animal production. Indigenous species of non-toxin producing anaerobic bacteria (Gram-positive, spore-forming *Clostridium* spp.) promote anti-inflammatory immune responses in the mammalian gut by activating T-regulatory cells and these bacteria make up a large proportion of the monogastric animal intestinal microflora [67]. Consequently, the genus *Clostridium* is a diverse group with many indigenous species that reside in the GI tract, several of which are known pathogens, but many are either benign or can be utilized as probiotics [68]. In fact, rational selection of an anaerobic, spore-forming mixture of bacteria utilizing chloroform extraction of mouse feces was completed to develop seventeen strains of spore-forming bacteria that can be orally administered to mice for attenuating colitis and allergic diarrhea [69]. Oakley et al. hypothesized that selecting spore-forming, non-toxin producing bacterial taxa closely related to known pathogens offers potential for competitive exclusion of pathogenic bacteria. Based on chloroform treatment [67, 69] of chicken gastrointestinal contents, axenic isolates were obtained that represent novel clostridia by 16S rRNA sequencing [70], several of which produced growth reduction on lawns of *C. perfringens*, *C. septicum* and *C. difficile* (Figure 3). The antimicrobial mechanisms are unknown, although several interesting genes such as potential prophage holins and endolysins were identified by sequencing genomes of the newly obtained isolates. Potential probiotics could also be identified from free-ranging species [71] and isolation of potential probiotic bacteria from a variety of free-ranging species could be of value for commercial food-animal production [72] with the minimal result being discovery of previously undiscovered bacteria. Investigations have been initiated for isolation of potential novel probiotic bacteria from Canada geese (*Branta canadensis*) feces with the hypothesis that enriching avian feces for chloroform-resistant bacteria will select for bacterial spore-formers that represent potentially non-toxin producing bacteria that could be utilized as probiotics for poultry or other birds. This has resulted in isolation of both anaerobic and aerobic, Gram positive and negative axenic cultures that can be screened for probiotic properties [73].

## Microbial-ecology driven discovery of antimicrobials from the environment

Antimicrobials can be discovered from a variety of different environments as natural products from uncultured bacteria as secondary metabolites. There has been a substantial decline in new drug approvals, however under-explored biological systems offer the potential for discovery for new natural product drug discovery [74–76]. The marine environment is a potentially abundant source of diverse natural products that include chemicals with antibacterial, antifungal, antiviral, antiparasitic, antitumour, anti-inflammatory, antioxidant, and immunomodulatory activities. Consequently, the diversity and abundance of natural products in marine environments could serve as a source of new therapies to treat drug-resistant infections [77].

Dr. Jason Kwan from the University of Wisconsin, USA has utilized microbial-ecology guided discovery of antibiofilm compounds from marine sponges. The approach is based on analyzing the largely unculturable bacteria associated with these filter-feeding invertebrates that have microbiomes that are implicated in the production of many compounds that may protect the sponge from predation. Many of the bacteria found among marine invertebrates’ complex microbiomes are phylogenetically divergent and lack genomes found in any reference databases [78]. Consequently, searching for new antimicrobial compounds can be accomplished via comparative shotgun metagenomics and metatranscriptomics to identify biosynthetic pathways that are upregulated in “perturbed” microbiomes that can be combined with chemical isolation to determine how the sponge microbiome protects itself from non-symbiotic bacteria. Dr. Kwan et al. have developed a custom bioinformatics pipeline that allows for assembly and separation of genomes among hundreds of bacteria from a single environmental metagenome. The resultant information is then utilized to direct efforts to culture antimicrobial-producing bacteria, or to clone and express pathways of interest for compound production in the lab. Using this approach, Dr. Kwan reported progress towards codon-optimized expression of chemical pathways, such as that for mandelalides that have been reported as potential antitumor agents as well as toxic for MRSA [79].

## Conclusions and future directions for development of microbial-derived antimicrobials

There is no doubt that antibiotics have been very successful in saving both human and animal lives, but those successes are now being challenged by the development of antibiotic-resistant bacteria. The use of antibiotics as growth promoters during food-animal production has come under increasing scrutiny as contributing to increased prevalence of bacteria resistant to many antimicrobials, such that the use of AGPs has been restricted or banned in many countries during food-animal production. Unfortunately, only a limited number of newly-developed antimicrobials have been developed for general use to treat bacterial [4, 80]. Certainly, alternatives to currently utilized antibiotics as previously stated should target individual pathogens to avoid overall reductions among other valuable components of the microbiome, stimulate positive immune responses such as T-regulatory cell development and be accompanied by increasing our knowledge of microbial communities.

A variety of approaches presented at the symposium included continued development of CRIPR/cas systems such that gene drive systems rely on bias in the targeted organism towards a specifically chosen locus and therefore offer an inherent specificity for a targeted pathogen. The principle challenge for the CRIPR/cas technology is application of the genome editing system so that it is delivered to at least 99% of a pathogen’s population upon treatment. This strategy is dependent on how to engineer phages to do this best and a pharmaceutical company is currently working on delivery mechanisms, but the data is not currently available for publication. Beyond bacteriophages, the Artilysins technology is currently being applied topically. If these or other technologies such as bacteriophage lysins are to be applied in animal feeds during production, it will require future investigations to determine if, following lysin treatment, toxins are released in virulent amounts by pathogens found in the GI tract, such as *C. perfringens*. The importance of research on microbial-derived products and the possibility of bringing any of the compounds discussed as a new animal health drug to market must pass to the regulatory stage. Since many of the producing organisms are commonly found in the environment, the hope is that evaluation and regulatory approval of new antimicrobial products will progress without major problems.

Research on characterizing environmental and animal microbiomes needs to be expanded because yeast and fungi are often under-studied, since many microbiome investigations rely heavily on analyses of 16S rRNA data. Also, the Archaea are often neglected for similar reasons [81]. Consequently, the search for probiotic organisms could be expanded to other microbial organisms that potentially include archival fecal or GI tract samples from agriculturally important animals along with free-ranging animals closely related to food-producing animals. There has been recent interest in utilizing Clostridia as probiotics and there is potential for considering these organisms as probiotics, but they could be difficult to produce since they are anaerobes. Clostridia are already on the market as probiotics for human gastrointestinal applications, so this is an opportunity for new investigations into potential use of clostridia as probiotics during food-animal production [82]. Finally, the in ovo methodology can be adopted to supply the chicken embryo with additional nutrients prior to hatching which will continue to be utilized by the chick post-hatch during the fasting period. Therefore, there is potential to utilize *in ovo* injection for establishing a healthy and diverse community of microorganisms to colonize the developing GIT that will provide both protection from pathogen invasion and improvement in growth performance to developing chicks [83–85].

The key issues addressed continue to be a need to further understand mechanism of action for alternatives to antimicrobials, to improve understanding of both food- and free-ranging animal microbiomes including understudied microbial organisms such as yeast or Archaea, and finally to address regulatory constraints and production issues such as cost.
